# A Ferroptosis-Related lncRNAs Signature Predicts Prognosis and Therapeutic Response of Gastric Cancer

**DOI:** 10.3389/fcell.2021.736682

**Published:** 2021-12-02

**Authors:** Shilang Xiao, Xiaoming Liu, Lingzhi Yuan, Fen Wang

**Affiliations:** ^1^ Department of Gastroenterology, The Third Xiangya Hospital, Central South University, Changsha, China; ^2^ Hunan Key Laboratory of Non-resolving Inflammation and Cancer, Changsha, China

**Keywords:** gastric cancer, ferroptosis, lncRNA, stromal, immune microenvironment, therapeutic response, immunotherapy

## Abstract

**Background:** Accumulating literature demonstrates that long noncoding RNAs (lncRNAs) are involved in ferroptosis and gastric cancer progression. However, the predictive value of ferroptosis-related lncRNAs for prognosis and therapeutic response is yet to be elucidated in gastric cancer (GC).

**Method:** The transcriptomic data and corresponding clinical information of GC patients were obtained from the Gene Expression Omnibus (GEO) and The Cancer Genome Atlas (TCGA) database. The association between ferroptosis-related lncRNAs and ferroptosis regulators was analyzed by Spearman correlation analysis. Then, we established a risk predictive model based on the ferroptosis-related lncRNAs using multivariate Cox regression analysis. Furthermore, we performed correlation analysis for the risk score and characteristics of biological processes, immune landscape, stromal activity, genomic integrity, drug response, and immunotherapy efficacy.

**Results:** We constructed a 17-ferroptosis-related-lncRNA signature via multivariate Cox analysis to divide patients into two groups: low- and high-risk groups. The low-risk group was linked to prolonged overall survival and relapse-free survival. The risk score had good predictive ability to predict the prognosis of GC patients compared with other clinical biomarkers. We found that the high-risk group was associated with activation of carcinogenetic signaling pathways, including stromal activation, epithelial-mesenchymal-transition (EMT) activation, and immune escape through integrated bioinformatics analysis. In contrast, the low-risk group was associated with DNA replication, immune-flamed state, and genomic instability. Additionally, through Spearman correlation analysis, we found that patients in the high-risk group may respond well to drugs targeting cytoskeleton, WNT signaling, and PI3K/mTOR signaling, and drugs targeting chromatin histone acetylation, cell cycle, and apoptosis regulation could bring more benefits for the low-risk group. The high-risk group was associated with poor immunotherapy efficacy.

**Conclusion:** Our study systematically evaluated the role of ferroptosis-related lncRNAs in t tumor microenvironment, therapeutic response, and prognosis of GC. Risk score–based stratification could reflect the characteristic of biological processes, immune landscape, stromal activity, genomic stability, and pharmaceutical profile in GC patients. The ferroptosis-related lncRNA signature could serve as a reliable biomarker to predict prognosis and therapeutic response of patients with GC.

## Introduction

Gastric cancer (GC) ranks in the top 10 of all malignancies worldwide with more than one million new diagnosed cases annually. Due to delayed diagnosis and treatment, GC is the third cause of cancer-related death with an estimated 78,000 deaths annually (Bray et al., 2018). For patients with advanced stage or distant metastatic adenocarcinoma, the 5-year overall survival (OS) rate decreases to approximately 5%, and the median survival is less than 1 year regardless of intensive treatments (Smyth et al., 2020; Thrift and El-Serag, 2020). There are only three biological biomarkers that can predict the therapeutic response of targeted therapy for GC patients: HER2 positivity for trastuzumab and trastuzumab deruxtecan, microsatellite instability (MSI) status, and PD-L1 expression for pembrolizumab (Nakamura et al., 2021). Hence, identifying novel biomarkers to predict prognosis and therapeutic response of GC patients is of clinical significance.

The low OS rate of GC patients is usually attributed to the high invasion ability of GC cells and resistance to current therapy. The latest study links ferroptosis to invasion, metastasis, and chemotherapy resistance of GC. However, the detailed role of ferroptosis is still unclear. This study aims to investigate the crucial role of ferroptosis in invasion and chemotherapy resistance and establish a ferroptosis-based signature to reflect the biological characteristics of individual GC patients.

Ferroptosis is a newly identified form of programed cell death, exhibiting distinct manifestations in morphology, biochemistry, and genetics in contrast to apoptosis and autophagy (Zhuo et al., 2020). The initiation and execution of ferroptosis are linked to the iron-mediated accumulation of reactive oxygen species and overproduction of lipid peroxidation (Stockwell et al., 2017). Increasing evidence demonstrates that ferroptosis involves multiple biological processes and therapeutic responses of gastric cancer patients. A study suggests that the downregulation of SLC7A11 targeted by MiR-375 attenuates the stemness of GC cells through triggering ferroptosis (Ni et al., 2021). Other research demonstrates that inhibiting ferroptosis decreases chemosensitivity of cisplatin and paclitaxel through the miR-522/USP7/hnRNPA1/ALOX15 axis (Zhang et al., 2020a). Besides this, Actinidia chinensis Planch, an antitumor drug, is also reported to inhibit the proliferation and migration of GC cells through induction of ferroptosis (Gao et al., 2020).

Long noncoding RNAs (lncRNAs) are defined as noncoding transcripts with a length longer than 200 nucleotides (Kim et al., 2021). Accumulating literature reveals that lncRNAs are involved in regulating multifarious biological processes of GC, such as DNA damage repairing, proliferation, immune escape, and drug resistance. For example, SNHG17, a type of lncRNA, shifted the double-strand break repair balance from homologous recombination toward nonhomologous end-joining in facilitating carcinogenesis upon *H. pylori* infection (Han et al., 2020). LncRNA UCA1 repressed expression of antitumor miRNA, including miR-26a/b, miR-193a, and miR-214, to facilitate proliferation, migration, and immune escape of GC (Wang et al., 2019a). Additionally, multiple lncRNAs in plasm or serum are demonstrated to possess outstanding ability to predict the prognosis of GC. For example, the expression of GASL1 is associated with the size, stage, and metastasis of GC (Yuan et al., 2020). The link between lncRNA and ferroptosis is also widely investigated. Recent research indicates that lncRNA OIP5-AS1 inhibits ferroptosis in prostate cancer through miR-128-3p/SLC7A11 signaling (Zhang et al., 2021). It is reported that lncRNA ZFAS1 could serve as competing endogenous RNAs to promote ferroptosis in patients with pulmonary fibrosis through the miR-150-5p/SLC38A1 axis (Yang et al., 2020). Despite the abundant studies focusing on lncRNA and ferroptosis, the role of ferroptosis-related lncRNA in predicting prognosis and therapeutic response is not comprehensively investigated in GC.

In this study, the role of ferroptosis-related lncRNAs in GC was comprehensively evaluated by integrating the TCGA and GEO databases. Besides this, we constructed a risk score model based on 17 ferroptosis-related lncRNAs. The predictive prognostic efficacy was compared between the risk score and other clinical biomarkers. Besides this, biological characteristics, immune landscape, stromal activity, and genomic integrity were compared between the low- and high-risk groups through a broad array of bioinformatic analyses. The purpose of our research was to identify a novel biomarker with excellent capability to predict prognosis and drug response of GC in clinics.

## Methods and Materials

### Data Collection and Processing

The gene expression data and corresponding clinical information were obtained from TCGA (n = 300) and GEO databases, including GSE62254 (n = 300), GSE84437 (*n* = 433), and GSE14549 (*n* = 192) cohorts. Finally, a total of 1275 GC patients were included in our study. The TCGA-STAD cohort was used as a training cohort to construct the risk model, and the microarray data from the GEO database was used as validation cohorts. For the TCGA-STAD cohort, we obtained RNA-sequencing data in raw count format from the UCSC Xena Browser (https://xenabrowser.net/datapages/) and normalized it using the Deseq2 package for further analysis. For microarray data from the Affymetrix platform, the raw “CEL” files of each cohort were downloaded and normalized using the adopted multiarray averaging method. For microarray data from other platforms, we directly downloaded normalized data from the GEO database. Besides this, the mutation information of GC patients was obtained from the TCGA database.

Additionally, we collected expression data and clinical information of the IMvigor210 cohort, an immunotherapy cohort, from http://research-pub.Gene.com/imvigor210corebiologies (Mariathasan et al., 2018). The expression data in raw count form was normalized using the DEseq2 package for further analysis.

### Identification of Ferroptosis-Related lncRNAs

Ferroptosis regulators, including 108 ferroptosis drivers and 69 ferroptosis suppressors, were obtained from the FerrDb website (Zhou and Bao, 2020). Besides this, based on the annotation file of lncRNA downloaded from the GENCODE website, the expression of 10,614 lncRNAs was retrieved from the TCGA database. Spearman correlation analysis was performed to analyze the relevance between lncRNAs and ferroptosis regulators. Ferroptosis-related lncRNA was defined as lncRNAs significantly related to at least one regulator (|spearman correlation| ≥ 0.2 and *p*-value < .05).

### Construction of Ferroptosis-Related Risk Model

First, the prognostic ferroptosis-related lncRNAs were selected through Univariate Cox regression analysis (*p* < .01). Then, candidate ferroptosis-related lncRNAs were selected through the least absolute shrinkage and selection operator (LASSO) analysis. Finally, stepwise multivariate Cox proportional hazard regression analysis was performed to establish a risk model and calculate risk score. The calculation formula of risk score is below:

$$Risk\;score=\;\overset{n}{\underset{i=1}{\sum }}Coef_{i}*Exp_{i}$$

The $Coef_{i}$ and $Exp_{i}$ represent the coefficient and expression levels of the corresponding lncRNA, respectively. The patients were classified into the low- or high-risk group based on the cutoff value of risk score dichotomized by “surv-cutpoint” function of the survminer package.

### Gene Set Variation Analysis and Functional Annotation

The gene set variation analysis (GSVA) was performed using the GSVA package to investigate the biological characteristics in different risk groups (Hänzelmann et al., 2013). The gene set used in the GSVA algorithm was “c2. cp.kegg.v7.2. symbols” downloaded from the MSigDB database. Functional annotation for differential expressed genes between the low- and high-risk groups was executed on the “Metascape” website (Zhou et al., 2019).

### Estimation of the Immune Landscape

The trait gene signatures for 28 types of immune cells were retrieved from Charoentong’s study (Charoentong et al., 2017). Single-sample gene-set enrichment analysis (ssGSEA) was performed to estimate the infiltration abundance of each distinct immune cell in each sample. The stromal score for each patient was calculated using the ESTIMATE algorithm in the estimate package (Yoshihara et al., 2013).

### Association Between Risk Score and Tumor-Related Biological Processes

The trait gene panels of biological processes related to cancers were obtained from Mariathasan’s study, including 1) EMT signatures, 2) angiogenesis signature, 3) pan-fibroblast TGFβ response signature (Pan-F-TBRS), 4) Wnt signaling, 5) mismatch repairing signature, and 6) cell cycle signature (Mariathasan et al., 2018). Additionally, the Asian Cancer Research Group (ACRG) constructed a series of gene panels, including MSI signature, epithelial-mesenchymal-transition (EMT) negative signature, EMT positive signature, and proliferation signature to identify the distinct molecular subtype of GC (Cristescu et al., 2015). In this study, we estimated enrichment score of the above gene signatures via the GSVA algorithm and compared it between the low- and high-risk groups (*p* < .05).

### Calculation of EMT Score

The EMT gene panel, including 25 epithelial markers and 52 mesenchymal markers, was obtained from Mak et al. (2016). The calculation formula of the EMT score is below.

$$EMT\;score=\;\sum_{i}^{N}\frac{M^{I}}{N}-\;\sum_{j}^{n}\frac{E^{j}}{n}$$

This calculation algorithm is demonstrated in a previous study, in which M and E represent the expression of the mesenchymal and epithelial genes, respectively, and N and n, respectively, represent the number of mesenchymal and epithelial genes.

### Validation of Ferroptosis-Related Risk Model

1) The differential expression analysis between the low- and high-risk groups was performed to identify gene signature A/B. Gene signature A is a panel of genes with higher expression in the high-risk group, and gene signature B exhibits higher expression in the low-risk group.
2) GSVA enrichment analysis was performed to calculate enrichment score of gene signature A/B in each sample. Then, a score termed an RS score, an alternative to the risk score, was equivalent to subtraction of the enrichment score of gene signature A from the enrichment score of gene signature B.
3) Through calculating the RS score in each GEO cohort, the Kaplan–Meier method and log-rank tests were performed to compare OS between the low- and high-RS score groups.

### The Potential Therapeutic Value of the RS Score

We downloaded the transcriptional expression data and drug response of more than 1000 cancer cell lines from Genomics of Drug Sensitivity in Cancer database (GDSC, http://www.cancerrxgene.org/downloads) (Yang et al., 2013). First, we calculated the RS score of each cell line. Then the Spearman correlation (Cor) between the RS score of each cell line and half-maximal inhibitory concentration (IC50) of each cell line to particular drugs was calculated. We considered | Cor | > 0.2 and *p* < 0.05 as significant correlations.

### Statistical Analysis

All statistical analysis and visualization were done in R 4.0.1 software. Spearman and distance correlation analyses were performed to quantify relevance among risk, RS, and EMT scores. Wilcoxon test or *t*-tests were performed to conduct difference comparisons of two groups. The cutoff value with maximum selected log-rank statistics of risk score and RS score were identified through the “surv-cutpoint” function of the survminer package. Based on the cutoff value, patients in each cohort were classified into the low- and high-risk groups. The Kaplan–Meier method and log-rank tests were performed to conduct a survival comparison between the low- and high-risk groups. We executed multivariate Cox analysis to assess robustness and independence of risk score. Receiver operating characteristic (ROC) and the area under the curve (AUC) were utilized to assess specificity and sensitivity of risk score or RS score using the Time ROC package. We depicted the mutation profile and calculated tumor mutation burden (TMB) using the maftool package (Mayakonda et al., 2018). In our study, two-sided *p* < .05 was considered as statistical significance.

## Results

### Identification of Ferroptosis-Related lncRNAs in GC Patients

The workflow of this study is summarized in Supplementary Figure S1A. The ferroptosis regulators, including 108 ferroptosis drivers and 69 ferroptosis suppressors, were retrieved from the FerrDb database focusing on genes, small molecules, and diseases related to ferroptosis. The expression of 177 ferroptosis regulators and 10,614 lncRNAs were obtained from the TCGA database. The definition of ferroptosis-related lncRNAs was set as lncRNAs significantly related to at least one regulator (|Spearman correlation| ≥ 0.2 and *p*-value < 0.05). Finally, a total of 6161 lncRNAs were discerned as ferroptosis-related lncRNAs. The detailed correlation information between lncRNAs and regulators is displayed in Supplementary Table S1.

### Construction of Risk Model Based on Ferroptosis-Related lncRNAs

**FIGURE 1:**
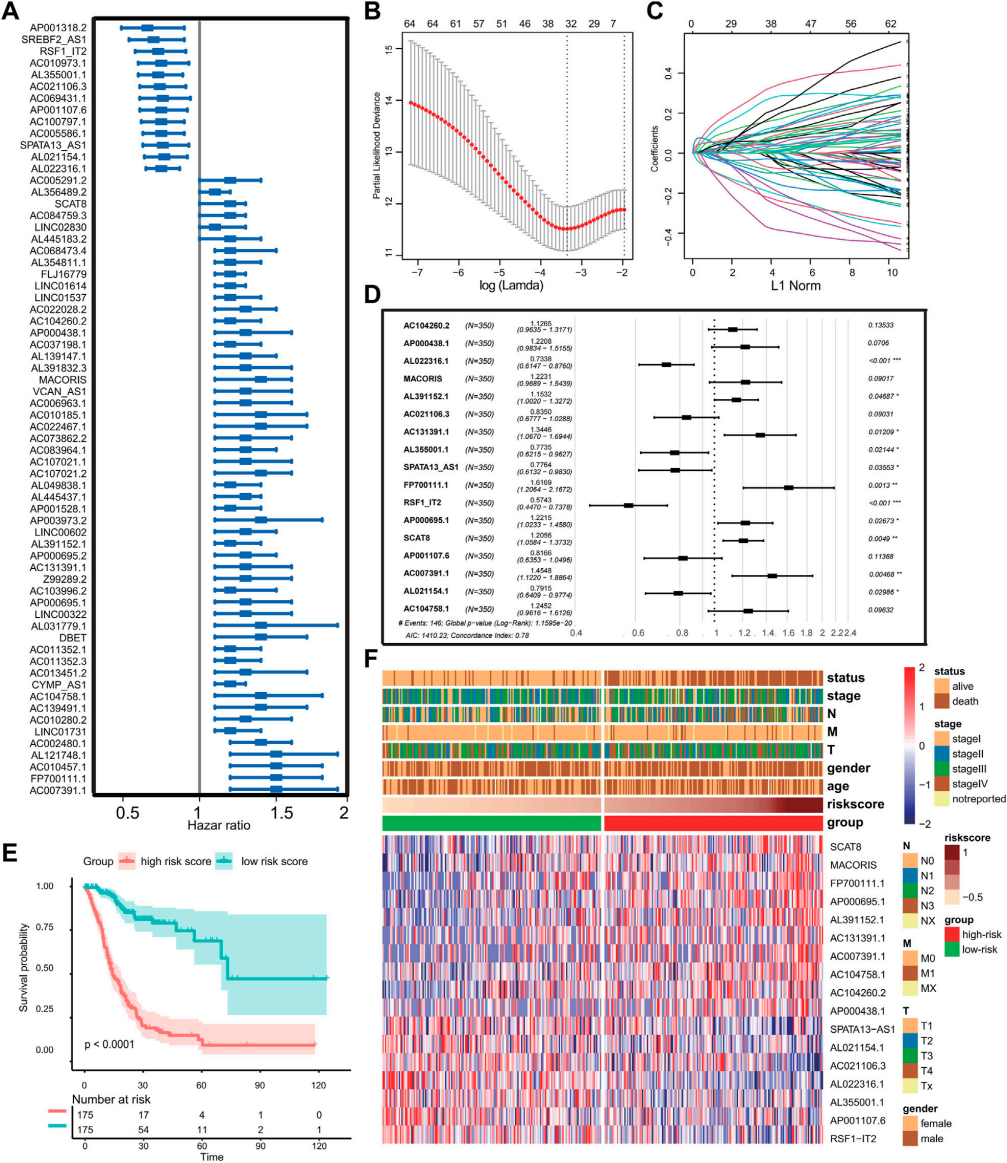
Construction of the prognostic ferroptosis-related lncRNA risk signature. **(A)** Forest plot showing prognostic ferroptosis-related lncRNAs selected by univariate Cox regression analysis. **(B–C)** LASSO regression analysis with minimum lambda value. **(D)** Forest plot exhibiting independently prognostic ferroptosis-related lncRNAs screened through stepwise multivariate Cox proportional hazard regression analyses. **(E)** Kaplan–Meier analysis and log-rank test for patients in the different risk groups. **(F)** Heat map displaying expression profile of prognostic ferroptosis-related lncRNAs and correlation between clinical features and risk score. (Ns represented no significance; *p < .05; **p < .01; ***p < .001; ****p < .0001).

First, univariate Cox regression analysis was performed to screen prognostic ferroptosis-related lncRNAs using *p*-value < 0.01 as the threshold. A total of 66 ferroptosis-related lncRNAs displayed significant correlations with the OS of GC patients (Figure 1A). Then, for enhancing predicting accuracy and avoiding overfitting, the LASSO-penalized Cox analysis was performed to sort out 31 ferroptosis-related lncRNAs with minimum lambda value (Figures 1B–C). Finally, stepwise multivariate Cox proportional hazard regression analysis was executed to screen 17 lncRNAs independently correlated with OS and construct a risk model to predict the prognosis of GC patients (Figure 1D). The patients in the TCGA cohort were categorized into two groups termed as the low- and high-risk groups based on the cutoff value of risk score provided by the surviminer package. The Kaplan–Meier method and log-rank tests show that patients in the high-risk group had worse OS compared with the low-risk group (*p* < 0.001) (Figure 1E). The expression landscape of 17 ferroptosis-related lncRNAs and the correlation between risk scores and clinical features including age, gender, TNM stage, grade, and survival status are shown in Figure 1F.

**FIGURE 2:**
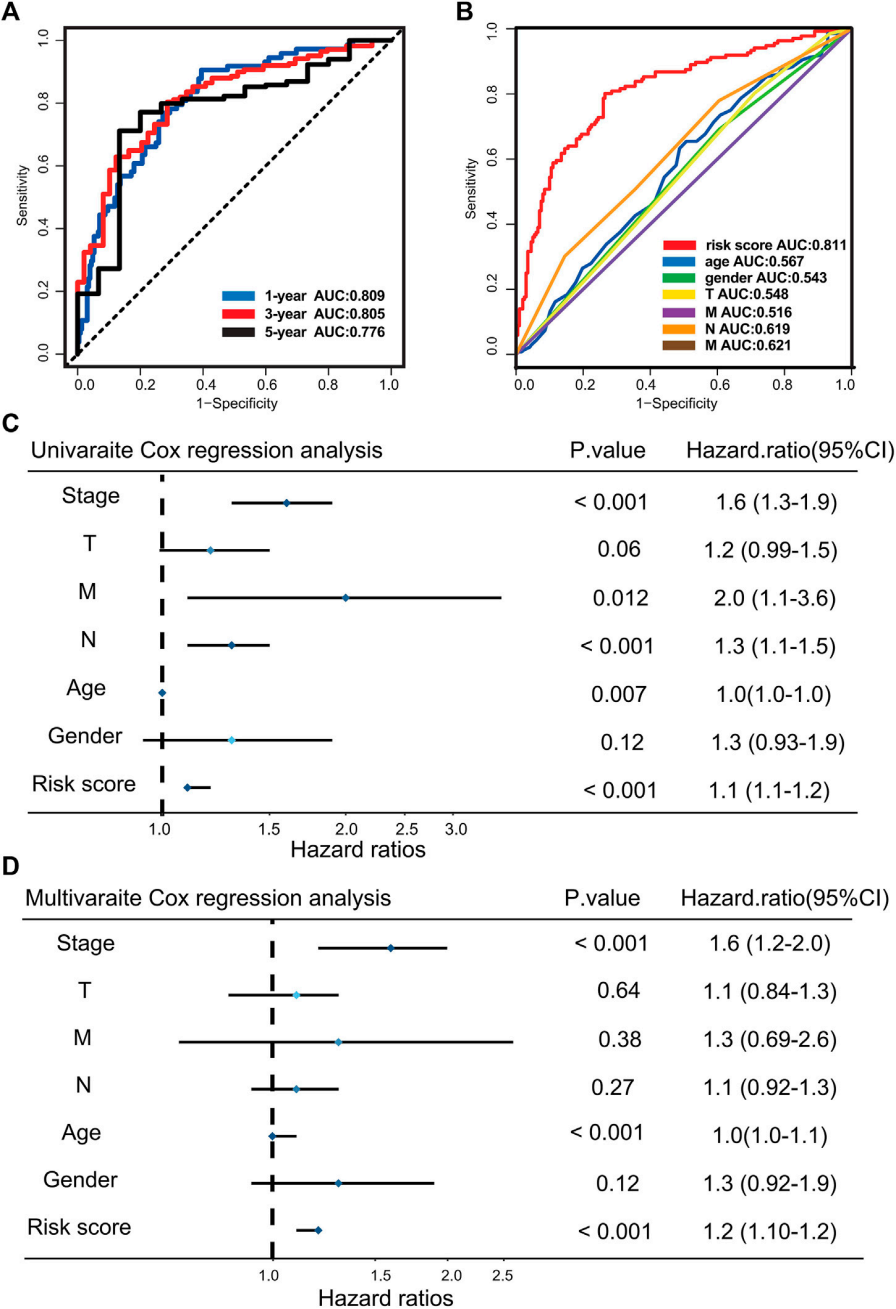
Evaluating the predictive efficacy of the risk model. **(A)** Time-dependent ROC curve of the risk score at 1, 3, and 5 years. **(B)** ROC curve for the risk score and clinical characteristics. **(C)** Univariate Cox analysis for clinical characteristics and the risk score with OS. **(D)** Multivariate Cox analysis for clinical characteristics and the risk score with OS.

The AUCs of time-dependent ROC curve for the risk score were 0.809, 0.815, and 0.776 at 1, 3, and 5 years, respectively (Figure 2A). The prediction accuracy of the risk score in the disease-specific survival (DSS), progression-free interval (PFI), and disease-free interval (DFI) survival were explored. The results of the Kaplan–Meier and ROC methods indicate that risk score possesses excellent capability to predict clinical outcome of GC patients (Supplementary Figure S1B–G). To further assess the uniqueness and susceptibility of the risk score in predicting the prognosis of GC patients, the AUC of risk score and other clinical factors such as age, gender, TNM, and stages were calculated. The results show that the AUC value of risk score was much higher than the AUC value of other clinical factors (Figure 2B), demonstrating that risk grade had a better prediction efficacy. Then, univariate Cox regression analysis and multivariate Cox proportional hazard regression analyses were executed to check whether the risk score could serve as an independent and robust biomarker to predict OS in GC patients. The hazard ratio of the risk score and 95% confidence interval were 1.1 and 1.1–1.2 (*p* < 0.001) in univariate Cox regression analysis, respectively (Figure 2C). In multivariate Cox analysis, the hazard ratio and 95% confidence intervals were 1.2 and 1.1–1.2 (*p* < 0.001), indicating that the prediction efficacy of risk score was not related to other clinical characteristics (Figure 2D). The above analysis demonstrates that the risk score is a reliable and robust biomarker to predict prognosis of GC patients.

### Biological Characteristics in Different Risk Score Groups

**FIGURE 3:**
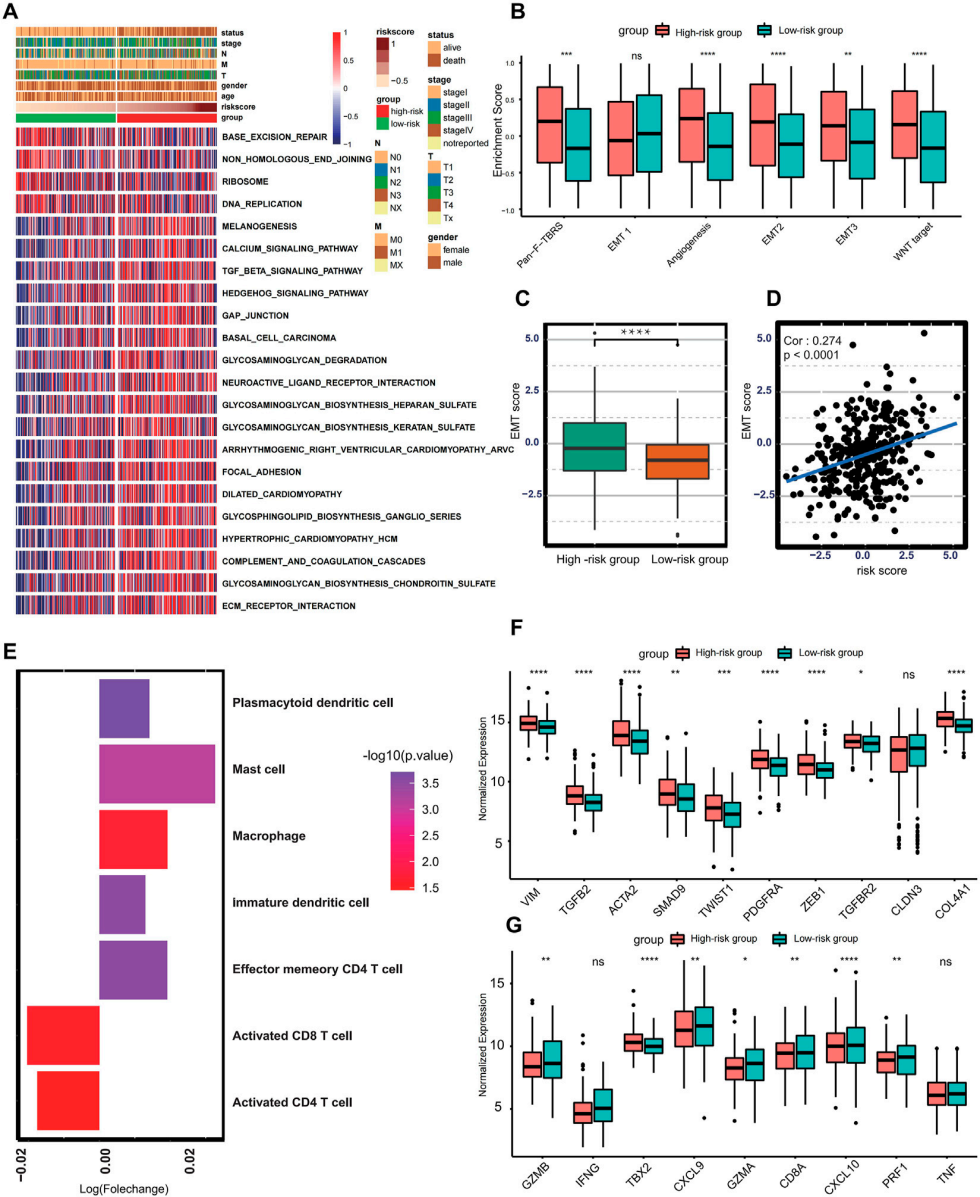
Biological characteristics in different risk score groups. **(A)** Heat map showing differentially enriched biological processes between the low- and high-risk groups. **(B)** Boxplot presenting different enrichment of EMT-related signaling between the low- and high-risk groups. **(C)** Box plot showing different EMT scores between the low- and high-risk groups. **(D)** Spearman correlation analysis between the risk score and EMT score. **(E)** Bar plot presenting different immune cell infiltration between the low- and high-risk groups. Immune cells with fold change >1 and *p* < .05 were enriched in the high-risk group, and those with fold change <1 and *p* < .05 were enriched in the low-risk group. **(F)** Boxplot showing expression of different molecules in TGF-beta or EMT pathway between the low- and high-risk groups. **(G)** Boxplot showing different expressions in transcripts of immune activation between the low- and high-risk groups. (Ns represents no significance; *p < .05; **p < .01; ***p < .001; ****p < .0001).

The GSVA pathway enrichment analysis was performed to investigate distinctive signaling pathways in different risk score groups. The high-risk group presented enrichment of carcinogenetic and stromal-related signaling, including TGF-beta pathway, Hedgehog pathway, ECM receptor interaction, and focal adhesion pathways. In contrast, the low-risk group presented enrichment pathways related to DNA replication and DNA damage repairing (Figure 3A). Previous studies pinpoint that activation of TGF-beta pathways plays a crucial role for the EMT, (Xu et al., 2009), indicating that risk score was associated with EMT to some extent. Subsequent enrichment analysis also shows that EMT-related signaling, and biological processes such as pan-F-TBRS, EMT2, EMT3, angiogenesis, and Wnt signaling were significantly enriched in the high-risk group (Figure 3B). Additionally, the EMT score of each GC patient was calculated based on the expression profile of epithelial markers and 52 mesenchymal markers. Compared with the low-risk group, patients in the high-risk group got higher EMT scores (*p* < 0.001) (Figure 3C). There was also a positive correlation between risk score and EMT score (Spearman correlation = 0.274 and *p*-value <0.001) (Figure 3D). Therefore, these results suggest that activation of EMT is a major biological characteristic of the high-risk group.

Currently, accumulating literature links ferroptosis to infiltrating immune cells within the tumor microenvironment (Tang et al., 2020). Therefore, the ssGSEA algorithm was performed to calculate the infiltration abundance of immune cells within the tumor microenvironment. The infiltration of CD8^+^ T cell and CD4+T cell was higher in the low-risk group, and tumors in the high-risk group present higher abundances of mast cells, plasmacytoid dendritic cells, macrophages, immature dendritic cells, and effector memory CD4^+^ T cells (Figure 3E). To better reveal the role of the risk score in the regulation of the tumor microenvironment, the cytokines and chemokines characterizing different risk groups were extracted from the published literature (Barbie et al., 2009; Zeng et al., 2019), for example, PDGFRA, TGFB2, SMAD9, TWIST1, CLDN3, TGFBR2, ACTA2, COL4A1, ZEB1, and VIM are correlated with the transcripts of TGF-beta or EMT pathway, and TNF, IFNG, TBX2, GZMB, CD8A, PRF1, GZMA, CXCL9, and CXCL10 are thought to be related to the transcripts of immune activation. The differential expression analysis reveals that almost all mRNA related to EMT processes were significantly higher in the high-risk group. In comparison, the low-risk group presents higher expression of mRNA involved in immune activation except for IFNG, TBX2, and TNF, demonstrating that the high-risk group is categorized as EMT-like subtype and the low-risk group is considered to be immune-activated phenotype (Figures 3F–G).

**FIGURE 4:**
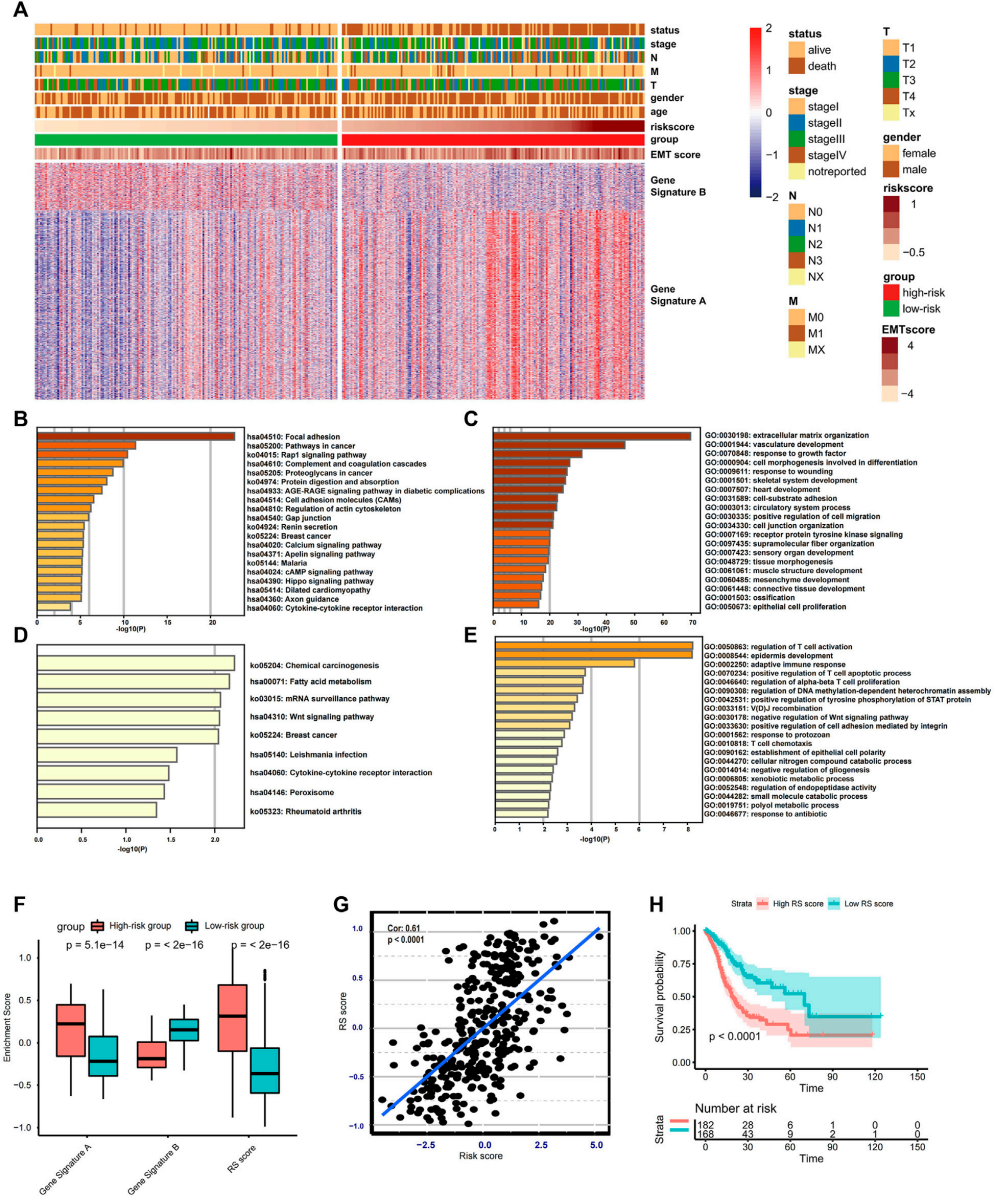
Construction and validation of the RS score. **(A)** Heat map displaying DEG signatures termed as gene signature A or B between the low- and high-risk groups. **(B–E)** KEGG and GO enrichment analysis for gene signature A or B. **(F)** Boxplot showing the difference in the enrichment score of gene signature A or B and the RS score between the low- and high-risk groups. **(G)** Spearman correlation analysis between RS score and risk score. **(H)** Kaplan–Meier analysis and log-rank test comparing the OS of patients with different RS scores.

To better understand the functional role of the risk score, two gene signatures termed gene signatures A and B were identified. Gene signature A is a set of genes with higher expression in the high-risk group, and those exhibiting lower expression are categorized into gene signature B. The distinct expression landscape of gene signature A/B is displayed by heat map (Figure 4A). The KEGG and GO enrichment analysis show that genes from gene signature A usually target stromal-related signaling or biological processes, including focal adhesion, extracellular matrix organization, and mesenchyme development (Figures 4B–C). In contrast, genes in gene signature B–regulated activation, apoptosis, the proliferation of T cells, and other immune-related biological processes, such as cytokine–cytokine receptor interaction and T cell chemotaxis (Figures 4D–E). Apart from the risk score, we also establish a novel score model named gene signature score (GS score), which is equivalent to subtraction of the enrichment score of gene signature A from the enrichment score of gene signature B. The distribution of GS score and the other two enrichment scores are significantly different between the low- and high-risk group (Figure 4F). Additionally, there is a highly positive correlation between GS score and risk score (Spearman correlation = 0.61, *p* < 0.001) (Figure 4G), indicating that GS score could be an alternative score model to risk score when risk score is not available to calculate. Additionally, patients with high RS scores were associated with worse clinical outcomes (Figure 4H).

### The Mutation Landscape in the High- and Low-Risk Score Groups

**FIGURE 5:**
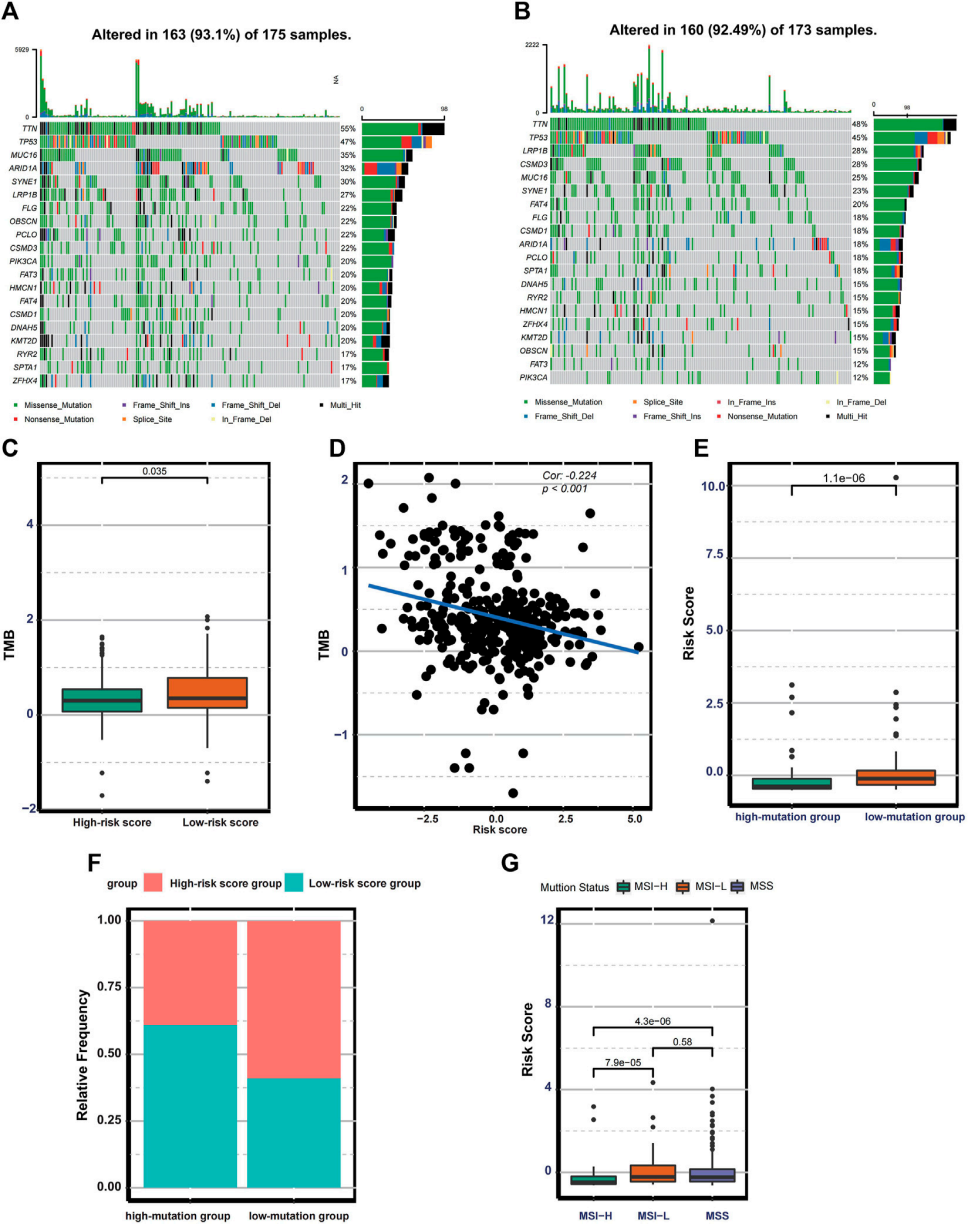
Mutation landscape in the different risk groups. **(A–B)** Waterfall plot displaying the mutation profile of genes with high mutation rates in the low- and high-risk groups. **(C)** Boxplot exhibiting the difference in TMB between the low-and high-risk groups. **(D)** Spearman correlation analysis between TMB and the risk score. **(E)** Boxplot exhibiting the risk score difference between the low-and high-mutation groups. **(F)** The bar plot displaying the relative distribution of different risk groups between the low- and high-mutation groups. **(G)** Boxplot showing the risk score differences among MSS, MSI-L, and MSI-H.

The distribution difference of somatic mutation between the low- and high-risk score groups was compared using the “maftool” package. The waterfall plot shows that the low-risk group presented a high genetic mutation rate (Figures 5A–B). Besides this, the low-risk group was remarkedly correlated with higher TMB in TMB quantification analysis (Figure 5C). We also confirm that there is a significantly negative correlation between TMB and risk score (Cor: 0.224, *p* < 0.001) (Figure 5D). In our study, patients with the top 25% of mutations in the TCGA cohort were considered as a high-mutation group, and patients with the bottom 25% of mutations belonged to a low-mutation group. The risk score quantification analysis demonstrates that the low-mutation group exhibited significantly higher risk scores (Figure 5E). A histogram of frequency distribution also indicates that patients in the high-risk score group are more likely to be classified into low-mutation groups (Figure 5F). Additionally, patients with MSI-H (microsatellite instability-high) subtypes exhibit lower risk scores compared with other two phenotypes with low microsatellite instability such as MSI-L and MSS (Figure 5G). These results indicate that the risk score based on 17 ferroptosis-related lncRNAs could reflect the genomic stability of patients with gastric cancer.

### Validating Prediction Efficacy of Risk Score

**FIGURE 6:**
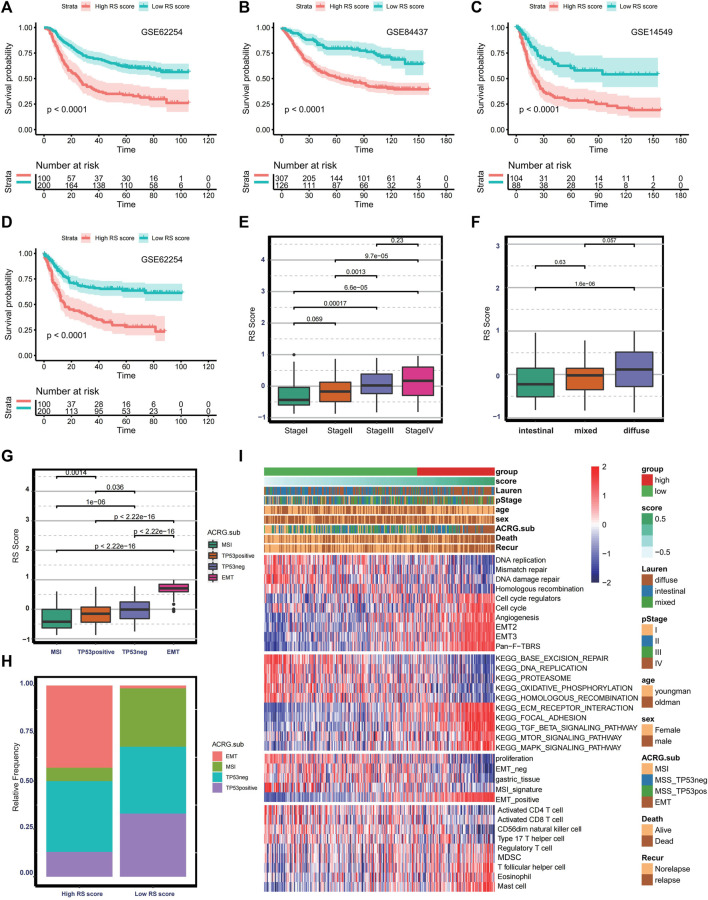
Validation of the prognostic ferroptosis-related lncRNA gene signatures. **(A–C)** Kaplan–Meier curve and log-rank test comparing OS in patients with low or high RS scores in GSE62254, GSE84437, and GSE14549. **(D)** Kaplan–Meier curve and log-rank test comparing relapse-free survival in patients with low or high RS score in GSE62254. **(E–G)** Boxplot comparing RS score in different stages, histological subtypes, and molecular subtypes in the GSE62254. **(H)** Bar plot presenting the relative frequency of four molecular subtypes between the low- and high- RS-score groups. **(I)** Heat map showing the difference in the biological characteristics and immune landscape between the low- and high- RS-score group.

Because the ferroptosis-related lncRNAs in the risk model were not detected in the microarray data, RS score was selected as an alternative indicator to validate the prediction efficacy of the risk score. Therefore, we investigated the prognostic value of RS score in GSE62254 (n = 300, log-rank test, *p* < 0.0001), GSE84437 (n = 433, log-rank test *p* < 0.0001), GSE14549 (n = 192, log-rank test *p* < 0.0001) (Figures 6A–C). The predictive value for relapse-free survival was proved in the GSE62254 (log-rank test *p* < 0.0001) (Figure 6D). The above analysis indicates that the risk score is a reliable prognostic indicator. Given that the GSE62254 provides comprehensive clinical information, we analyze the correlation between RS score and clinical characteristics. In the GSE622454 cohort, patients with advanced GC (stage III/IV) were associated with higher RS scores (Figure 6E). Patients with a diffuse histological subtype exhibit a higher RS score (Figure 6F), demonstrating that tumors with high RS score were significantly associated with high malignancy and rapid progression. Besides this, the EMT subtype presented the highest RS score, and the MSI subtype exhibited the lowest RS score among four consensus molecular subtypes (Figure 6G). The distribution of molecular subtypes between the low and high RS score groups was significantly different (Figure 6H). Patients with the EMT subtype were more likely to be classified into the high RS score group, and patients with the MSI subtype were more linked to the low RS score group. Similar to the risk score–based stratification, GSVA and ssGSEA analysis also confirmed EMT-related signaling pathways were significantly enriched in the high RS score group. In contrast, the low RS score group was associated with higher infiltration of CD8+T cells and CD4+T cells, higher enrichment of MSI signature, DNA replication, and DNA damage repairing signaling (Figure 6I). The high coincidence of tumor microenvironment trait and biological characteristics between the two risk models further demonstrate that RS score is a promising alternative to the risk score.

### The Role of Ferroptosis-Related Risk Score in Immunotherapy

**FIGURE 7:**
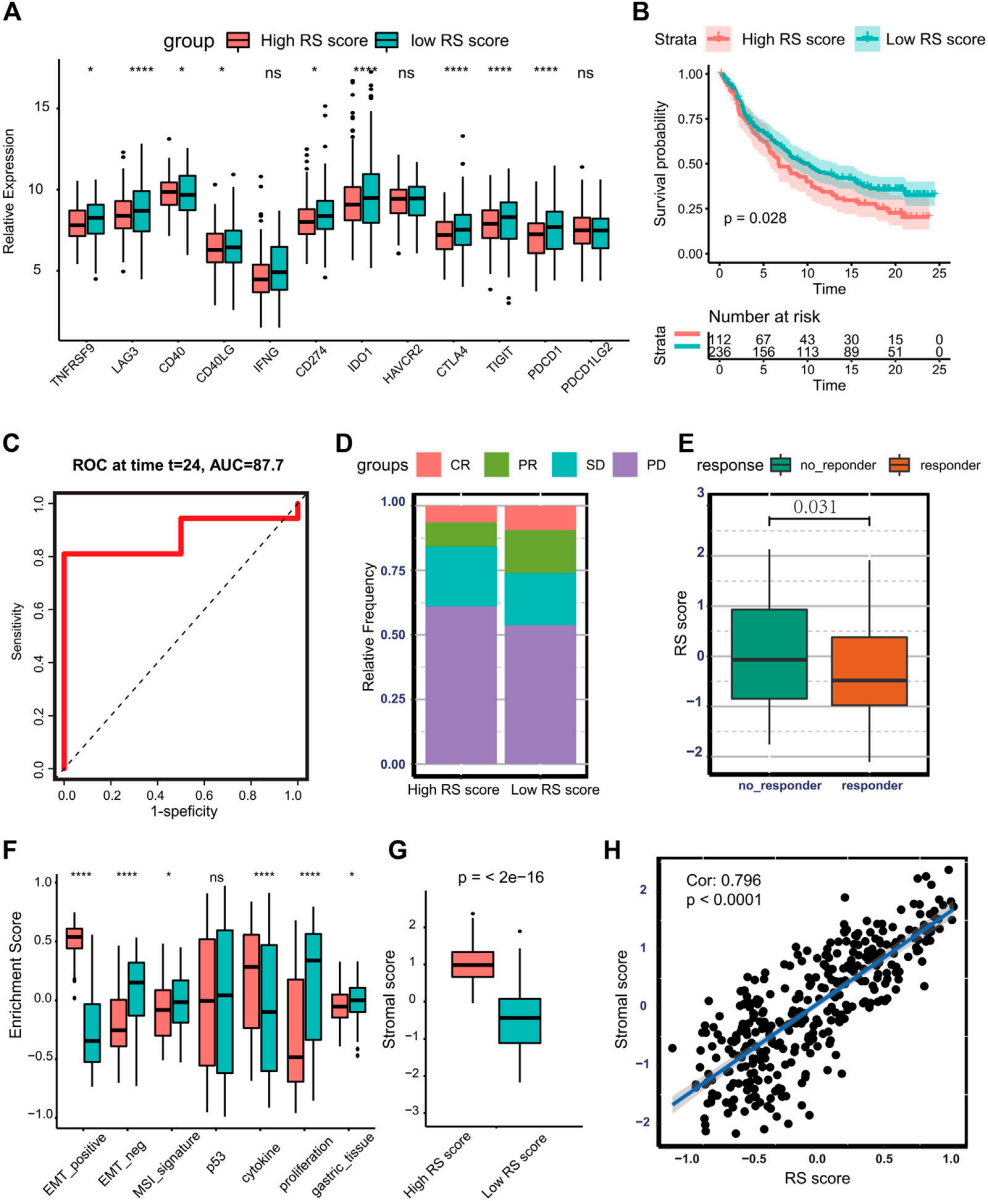
Predicting the therapeutic response of RS score to immunotherapy. **(A)** Boxplot presenting the expression of multiple checkpoints between the low- and high-RS score groups. **(B)** Kaplan–Meier curve and log-rank test compare the OS of patients with low or high RS scores in the IMvigor210 cohort. **(C)** Time-dependent ROC curve for RS score at 2 years in IMvigor210 cohort. **(D)** Bar plot displaying the relative frequency of different clinical response subgroups in the low or high RS score group. **(E)** Boxplot demonstrating the RS score difference between response group and no-response group. **(F)** Boxplot showing different activation degrees of gene signatures characterized by ACRG molecular subtype in the low and high RS score groups. **(G)** Boxplot presenting stromal score difference in the low and high RS score groups. **(H)** Spearman correlation analysis between stromal score and RS score in IMvigor210 cohort. (Ns represented no significance; *p < .05; **p < .01; ***p < .001; ****p < .0001).

Currently, increasing efforts are being made to discover novel biomarkers to predict immunotherapy efficacy, including TMB and expression of PD-L1. In the TCGA-STAD cohort, multiple immune checkpoints (Zhang et al., 2020b), including TNFRSF9, LAG3, CD40, CD40LG, CD274, IDO1, CTLA4, TIGIT, and PDCD1 are significantly higher in the low-risk group (Figure 7A). Therefore, we investigate whether the risk score could predict response to immunotherapy based on the anti-PD-L1 cohort (IMvigor210). To conduct this, patients in the IMvior210 cohort were assigned RS scores. It was found that patients with low RS scores had prolonged survival time compared with patients with high RS scores (log-rank test *p* = 0.028) (Figure 7B). The AUC of the time-dependent ROC curve for the RS score reached 0.877 at 2 years (Figure 7C). The response degree to immunotherapy was classified into four categories, including complete response (CR), partial response (PR), stable disease (SD), and progressive disease (PD). The objective response rate was significantly higher in the low RS score group than in the high RS score group (Figure 7D). Also, the no-response group presented higher RS scores (Wilcoxon test: *p* = 0.031) (Figure 7E). Collectively, the above results indicate that RS score is correlated with response to immunotherapy. Additionally, tumors with high RS scores exhibit higher enrichment scores of EMT-positive signature, and low RS score groups are associated with MSI signature and proliferative signature in the IMvior210 cohort (Figure 7F). Besides this, patients with high RS scores exhibited pronounced elevation of stromal score (Figure 7G). Moreover, there was a significantly positive correlation between stromal score and RS score (Cor: 0.796, *p* < 0.0001) (Figure 7H). The above analysis indicates that stromal activation is part of the causation of immunotherapy resistance.

### The Potential Therapeutic Value of RS Score

**FIGURE 8:**
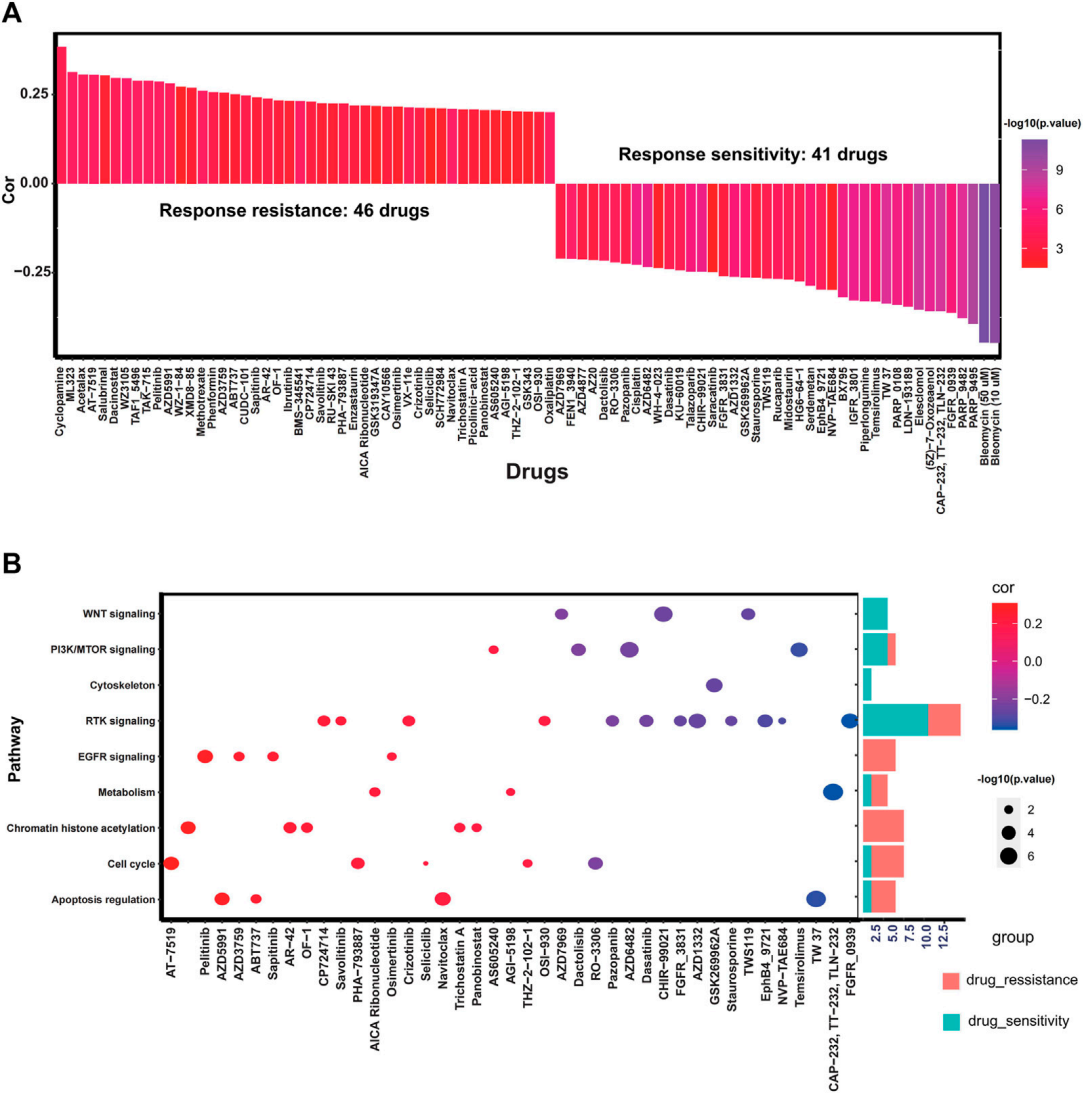
Potential predictive therapeutic value of the RS score. **(A)** Bar plot exhibiting Spearman correlation between RS score and drug sensitivity. **(B)** Dot plot and corresponding bar plot demonstrating the pathways targeted by drugs whose sensitivity is associated with the RS score.

To explore the effect of RS score on drug response, Spearman correlation analysis was performed to calculate the association between RS score of cell lines and half-maximal inhibitory concentration (IC50) of cell lines to specific drugs. A total of 87 drugs were correlated with the RS scores. Among them, 41 drugs exhibited drug sensitivity associated with the RS score, including DNA replication inhibitor Bleomycin (Cor: 0.44, *p* < 0.0001), RTK signaling inhibitor FGFR_0939 (Cor: 0.36, *p* < 0.001) and PI3K/mTOR signaling inhibitor Temsirolimus (Cor: 0.33, *p* < 0.001), and 46 drugs presented drug resistance associated with RS score such as Cyclopamine (Cor: 0.38, *p* < 0.001), Dacinostat (Cor: 0.30, *p* < 0.001), and AT-7519 (Cor: 0.31, *p* < 0.001). Other drugs associated with RS score are summarized in Figure 8A and Supplementary Table S2. The pathways targeted by these drugs are depicted in Figure 8B. We find that drugs whose sensitivity was correlated with high RS score usually targeted cytoskeleton, WNT signaling, and PI3K/mTOR signaling, and drugs related to low RS score were more likely to target chromatin histone acetylation, cell cycle, and apoptosis regulation. The above results indicate that the RS score might be a reliable biomarker to predict the drug response of individual GC patients.

## Discussion

Ferroptosis is a type of programed cell death (RCD), exhibiting distinct manifestation in terms of morphology, biochemistry, and genetics compared with apoptosis, necrosis, and other types of cell death. It is considered to be a promising antitumor target and is reported to regulate the progression of multiple cancers (Friedmann Angeli et al., 2019; Chen et al., 2020a). Additionally, multiple biomarkers with robust prediction efficacy for prognosis and antitumor therapy have been established based on ferroptosis (Liu et al., 2020; Zhuo et al., 2020). However, previous studies primarily focus on the protein-coding genes involved in the regulation of ferroptosis. Given that a wealth of literature demonstrates that epigenetic effectors such as lncRNA participate in the regulation of ferroptosis (Wang et al., 2019b), a comprehensive investigation of prognostic significance of ferroptosis-related lncRNAs is essential in GC.

In our study, 33 prognostic ferroptosis-related lncRNAs were selected through univariate Cox and LASSO regression analysis. Then, 17 of them were used to construct the risk-score model. In the TCGA-STAD cohort, the low-risk group exhibited a better OS, DSS, PFI, and DFI. Compared with other clinical biomarkers, such as TNM and stage, the risk score possesses a good predictive efficacy of prognosis. The biological characteristics, immune landscape, stromal activity, and genomic integrity were significantly different between the low- and high-risk groups through bioinformatic analysis. For expanding the application scope of the risk score, RS score was established based on gene signature A/B obtained from the differential analysis between the low- and high-risk groups. Similarly, the RS score showed reliable capacity in predicting clinical outcomes in multiple validation cohorts. Finally, we uncovered differential pharmacological profile and immunotherapy remission rates between the low and high RS score groups, indicating that risk score–based stratification could herald chemotherapy and immunotherapy therapeutic efficacy.

A panel of epigenetic effectors is closely linked to the induction of ferroptosis and tumorigenesis. As an essential regulator of epigenetic modification, the role of lncRNA in ferroptosis and tumor progression has been gradually excavated. For instance, lncRNA TINCR promoted trastuzumab resistance and EMT through facilitating the release of HER-2 and upregulating Snail-1 in breast cancer (Dong et al., 2019); LINC00336, a nuclear lncRNA, served as an endogenous sponge of microRNA 6,852 to inhibit ferroptosis (Wang et al., 2019b). Another study suggests that upregulation of cytosolic lncRNA P53RRA caused nuclear sequestration of p53, resulting in persistent activation of the p53 pathway and induction of ferroptosis (Mao et al., 2018). However, the role of lncRNA in ferroptosis remains mysterious, especially for GC. There is no report on using lncRNAs in a risk model to associate ferroptosis and tumorigenesis in GC. Our study reveals the role of these lncRNAs in ferroptosis and progression of tumor, providing a significant reference for future in-depth experimental studies. Interestingly, in lung cancer, RSF1-IT2 functions as a ceRNA to sponge miR-129-5p targeting SNAI1 and HMGB1, facilitating tumor metastasis and invasion (Wu et al., 2020). Nevertheless, RSF1-IT2 is linked to better clinical outcomes in the TCGA-STAD cohort. This contrasting phenomenon may be caused by tissue heterogeneity and other biological processes regulated by RSF1-IT1.

Increasing evidence demonstrates that ferroptosis is associated with tumor invasion and metastasis. A study of Jessalyn suggests that lymph fluid with high levels of glutathione and less free iron could increase their ability to form metastatic tumors through inhibiting ferroptosis (Ubellacker et al., 2020). Tumor plasticity, stemness, invasion, and metastasis are closely associated with EMT status (Pastushenko and Blanpain, 2019). Other research demonstrates that β-elemene, a ferroptosis inhibitor, could lower the expression of mesenchymal markers and upregulate expression of epithelial markers (Chen et al., 2020b). In our study, the pathway enrichment analysis reveals that the high-risk group had high expression of EMT markers and activation of EMT-related pathways such as TGF-beta signaling. Besides this, the risk score exhibits a significantly positive correlation with EMT score. Our results suggest that the high-risk group is an EMT-subtype with increasing plasticity, stemness, invasion, and metastasis capacity. The occurrence of EMT may partly explain the poor clinical outcomes of the high-risk group. Because plentiful damage-associated molecular patterns (DAMPs) could be released during cell death, ferroptosis is considered to be immunogenic cell death and could shape the immune landscape within thhe tumor microenvironment to some extent (Sun et al., 2020). In our study, The DEGs between the low- and high-risk groups usually targeted immune-related signaling, such as regulation for activation, apoptosis, and proliferation of T cells, cytokine–cytokine receptor interaction, and T cell chemotaxis. The low-risk group exhibited an abundance of activated CD8+T cells and activated CD4+T cells and upregulated expression of multiple immune chemokines, including GZMB, CD8A, PRF1, GZMA, CXCL9, and CXCL10. Although the high-risk group presented high infiltration of multiple innate immune cells such as plasmacytoid dendritic cells and macrophages, stromal activation in the high-risk group would cause immune cells to be excluded from parenchyma and trapped in stroma, resulting in the incapability of immune cells (Salmon et al., 2012; Joyce and Fearon, 2015). These discoveries indicate that the low-risk group ias in a relative immune-activated state. Additionally, the ferroptosis process is accompanied by vast production and accumulation of reactive oxygen species (ROS), causing frameshift mutation and microsatellite instability (Granofszky et al., 2018). Microsatellite instability augments neo-antigen load, resulting in increased immunogenicity and appealing more immune cells to tumor parenchymal (Shin et al., 2019). In our study, we found that the risk score or RS score was significantly higher in the MSI-H or MSI subtype, respectively. TMB was pronounced in the low-risk group and negatively correlated with the risk score. These results imply that the occurrence of MSI is another critical cause for the immune-flamed tumor microenvironment in the low-risk group.

There is emerging evidence that ferroptosis is involved in cancer immunotherapy. The study of Wang reports that immunotherapy-activated CD8+T cells could promote ferroptosis via IFNγ/SLC2A11 signaling, contributing to the antitumor efficacy of immunotherapy (Wang et al., 2019c). Considering the marked differences of the tumor microenvironment landscape between the low- and high-risk groups, we speculated that the risk score–based risk stratification could herald therapeutic efficacy of immunotherapy. In addition, the different expressional profiles of checkpoints between the low- and high-risk groups also support the above assumption. In the IMvior210 cohort, patients with low RS scores had a prolonged survival time, implying that the checkpoint blockade was an excellent regimen for patients in the low-risk group. Through GSVA enrichment analysis, the high-risk group was closely linked to the EMT subtype with activation of EMT-related signatures and high stromal score. Previous studies also demonstrate that mesenchymal tumor cells could define the tumor microenvironment by regulating the extracellular matrix and promoting T cell exclusion from tumors, facilitating acquired resistance to immune checkpoint blockades (Horn et al., 2020). Therefore, the dedifferentiation of tumor cells plays an essential role in resistance to immunotherapy.

Currently, chemotherapy is the foremost treatment for advanced GC, and drug resistance is a critical reason causing treatment failure and cancer-related death. Recent studies highlight the linkage between ferroptosis and chemoresistance. Zhang’s study demonstrates that cancer-associated fibroblasts secrete miR-522 to inhibit ferroptosis through suppressing ALOX15 and decreasing the accumulation of lipid peroxidation, ultimately leading to chemoresistance (Zhang et al., 2020a). Sun’s research also reveals that Metallothionein-1G facilitated sorafenib resistance through inhibition of ferroptosis (Sun et al., 2016). In our study, the high-risk group responded well to drugs targeting the cytoskeleton, WNT signaling, and PI3K/mTOR signaling, and drugs targeting chromatin histone acetylation, cell cycle, and apoptosis regulation could bring more benefits for the low-risk group. The previous study also identifies GC subtypes with differing drug responses. For example, tumor cells with a mesenchymal subtype were susceptible to PI3K/AKT/mTOR inhibitors, including ZSTK474 and GSK690693 (Lei et al., 2013). Although responses of ZSTK474 (Cor: 0.199, *p* = 0.002) and GSK690693 (Cor: 0.194, *p* < 0.0001) did not reach a significant correlation with the RS score, it was interesting that the high-risk group with EMT features was also sensitized to PI3K/mTOR inhibitors, suggesting that the risk score–based stratification had the reliable capability to predict therapeutical response of individual GC patients.

Considering the crucial role of ferroptosis in modulating biological behaviors of tumor cells and profound modulation of lncRNA in ferroptosis, risk models based on ferroptosis-related lncRNA have been constructed in multiple cancer types. Nevertheless, most studies mainly focus on the risk model’s prognostic value rather than its underlying mechanism and other application scope. Our study finds that the prediction efficacy of our risk model is attributed to its incredible ability to reflect stromal activation, TME landscape, genomic instability, and mutation landscape. In addition, we find that the risk score–based stratification could guide personal chemotherapy and immunotherapy for individual tumors, improving the prognosis of GC patients.

Inevitably, there are some limitations in our study. First, because few lncRNAs were examined in the microarray data, we had to use RS score other than risk score to validate the robustness and effectiveness of the prognostic model. Although the RS score exhibited a highly positive correlation with the risk score, the validation was indirect. Second, the detailed mechanisms between ferroptosis-related lncRNAs and ferroptosis, between ferroptosis and tumor microenvironment, and between ferroptosis and chemoresistance are still unclear. Further in-depth studies are required to explore the above interactions. Third, the establishment and validation of the risk model were based on the public databases, and using a prospective GC cohort could provide more evidence for assessing its clinical utility.

In conclusion, the risk model constructed in our study is a reliable and robust marker to predict the survival outcome of GC patients. Besides this, the risk score–based stratification is associated with different biological processes, immune landscape, stromal activity, and genomic stability. Also, the risk stratification provides promising clinical evidence to guide treatment regimens for GC patients. Our research further investigates the role of ferroptosis-related lncRNAs in the tumor microenvironment, pharmaceutical landscape, and prognostic prediction in GC, providing a novel insight for future research and clinical practice.

## Data Availability

The datasets presented in this study can be found in online repositories. The names of the repository/repositories and accession number(s) can be found in the article/Supplementary Material.

## Abbreviations

lncRNAs, Long noncoding RNAs; GC, gastric cancer; GEO, Gene Expression Omnibus; TCGA, The *Cancer* Genome Atlas; EMT, epithelial-mesenchymal-transition; MSI, microsatellite instability; GSVA, gene set variation analysis; ssGSEA, Single-sample gene-set enrichment analysis; Pan-F-TBRS, pan-fibroblast TGFβ response signature; ACRG, Asian *Cancer* Research Group; MSI, Microsatellite-instability; GDSC, Genomics of Drug Sensitivity in *Cancer* database; Cor, correlation; IC50, half maximal inhibitory concentration; ROC, Receiver operating characteristic; AUC, area under the curve; TMB, tumor mutation burden; CR, complete response; PR, partial response; SD, stable disease; PD, progressive disease; OS, overall survival; DSS, disease-specific survival; PFI, progression-free interval; DFI, disease-free interval.
