# Supplementary material for: A Ferroptosis-Related lncRNAs Signature Predicts Prognosis and Therapeutic Response of Gastric Cancer
Source: Front Cell Dev Biol. 2021 Dec 2;9:736682. doi: 10.3389/fcell.2021.736682 (PMC8674955; doi:10.3389/fcell.2021.736682)
Supplement: Supplementary file 1 [file DataSheet1.PDF]

Figure S1

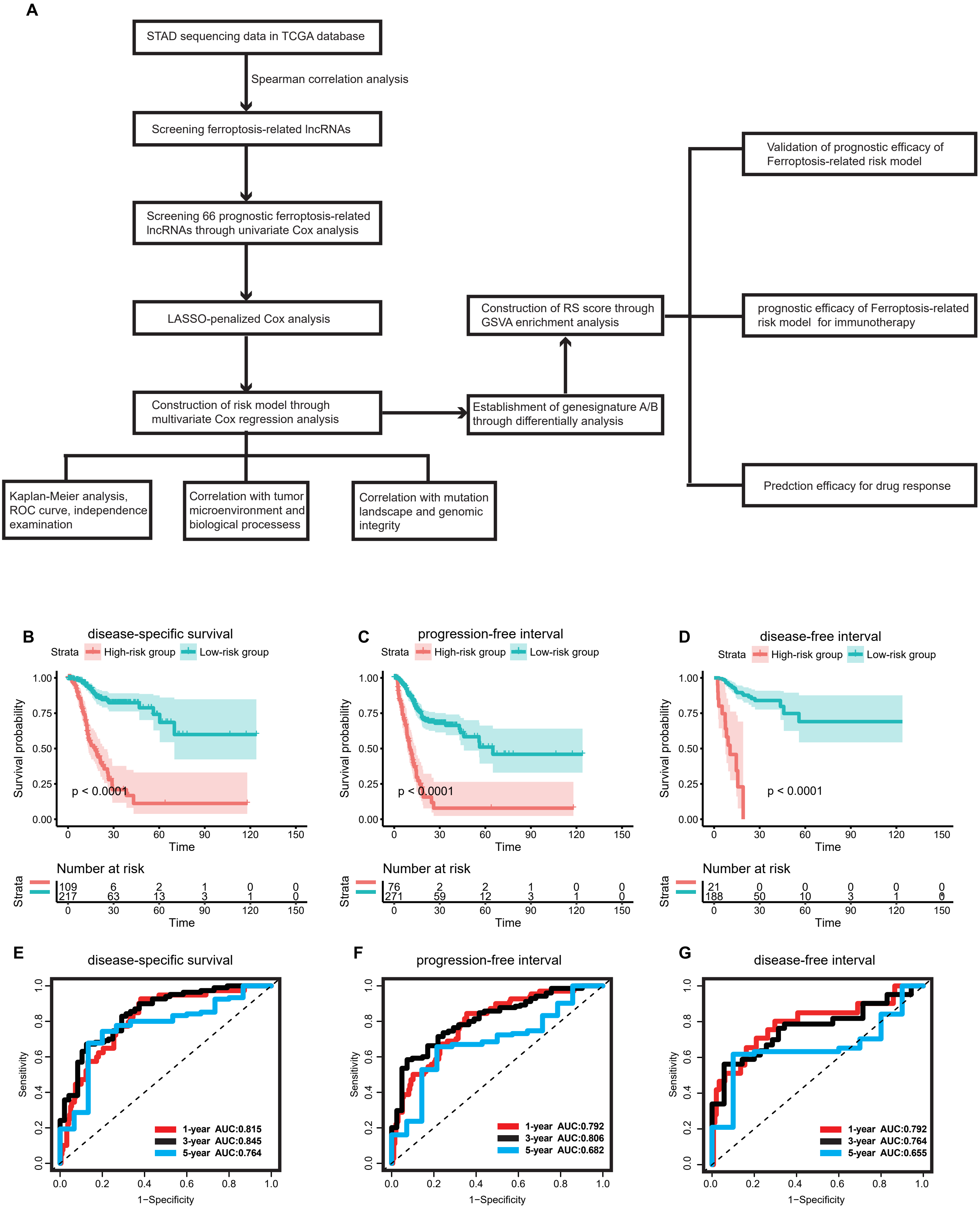
A
STAD sequencing data in TCGA database
Spearman correlation analysis
Screening ferroptosis-related lncRNAs
Screening 66 prognostic ferroptosis-related lncRNAs through univariate Cox analysis
LASSO-penalized Cox analysis
Construction of risk model through multivariate Cox regression analysis
Kaplan-Meier analysis, ROC curve, independence examination
Correlation with tumor microenvironment and biological processess
Correlation with mutation landscape and genomic integrity
Establishment of genesignature A/B through differentially analysis
Construction of RS score through GSVA enrichment analysis
Validation of prognostic efficacy of Ferroptosis-related risk model
prognostic efficacy of Ferroptosis-related risk model for immunotherapy
Predction efficacy for drug response
B
disease-specific survival
C
progression-free interval
D
disease-free interval
Strata
High-risk group
Low-risk group
Survival probability
p < 0.0001
Time
Number at risk
E
disease-specific survival
1-year AUC:0.815
3-year AUC:0.845
5-year AUC:0.764
F
progression-free interval
1-year AUC:0.792
3-year AUC:0.806
5-year AUC:0.682
G
disease-free interval
1-year AUC:0.792
3-year AUC:0.764
5-year AUC:0.655
Sensitivity
1−Specificity
